# Mechanistic Pathways for Peptidoglycan O-Acetylation and De-O-Acetylation

**DOI:** 10.3389/fmicb.2018.02332

**Published:** 2018-10-01

**Authors:** David Sychantha, Ashley S. Brott, Carys S. Jones, Anthony J. Clarke

**Affiliations:** Department of Molecular and Cellular Biology, University of Guelph, Guelph, ON, Canada

**Keywords:** peptidoglycan, O-acetylation, *O*-acetyltransferase, *O*-acetylesterase, SGNH hydrolase, catalytic mechanism, X-ray structure

## Abstract

The post-synthetic O-acetylation of the essential component of bacterial cell walls, peptidoglycan (PG), is performed by many pathogenic bacteria to help them evade the lytic action of innate immunity responses. Occurring at the C-6 hydroxyl of *N*-acetylmuramoyl residues, this modification to the glycan backbone of PG sterically blocks the activity of lysozymes. As such, the enzyme responsible for this modification in Gram-positive bacteria is recognized as a virulence factor. With Gram-negative bacteria, the O-acetylation of PG provides a means of control of their autolysins at the substrate level. In this review, we discuss the pathways for PG O-acetylation and de-O-acetylation and the structure and function relationship of the *O*-acetyltransferases and *O*-acetylesterases that catalyze these reactions. The current understanding of their mechanisms of action is presented and the prospects of targeting these systems for the development of novel therapeutics are explored.

## Introduction

Antimicrobial resistance (AMR) among bacterial pathogens continues to pose a serious threat to human health despite extensive research and altered clinical practices. In 2015, the World Health Assembly of the United Nations (UN) endorsed a global action plan to tackle AMR (World Health Organization, 2015), and the UN General Assembly met in 2016 to commit to it. This represented only the fourth time in the history of the UN that a health topic was discussed at the General Assembly, underscoring the importance of the issue. Indeed, an extensive review on AMR recently conducted for the UK government (The Review on Antimicrobial Resistance chaired by Jim O’Neill, 2014) suggests that, despite the huge resources that have already expended to date, a continued heavy investment into pharmaceutical research is needed to defend against the threat of drug-resistant “superbugs,” such as methicillin-resistant *Staphylococcus aureus* (MRSA), vancomycin-resistant *Enterococcus* (VRE), and drug-resistant *Streptococcus pneumoniae* (DRSP). As the Gram-positive pathogens MRSA, VRE, and DRSP continue to burden public health, AMR strains of the Gram-negative pathogen *Neisseria gonorrhoeae* are now emerging (Allen et al., 2014; Martin et al., 2015) to the extent that, in the summer of 2017, the WHO began warning of the spreading of drug-resistant gonorrhoea where some strains may be untreatable by all known antibiotics (World Health Organization, 2017). With the declining impact of traditional antibiotics, and few prospects in the development pipeline, alternative strategies are being considered (Brown and Wright, 2016). One of these concerns the development of antivirulence agents as antibiotics which would increase the susceptibility of pathogens to the host immune response while minimizing deleterious effects on commensal bacteria.

**FIGURE 1:**
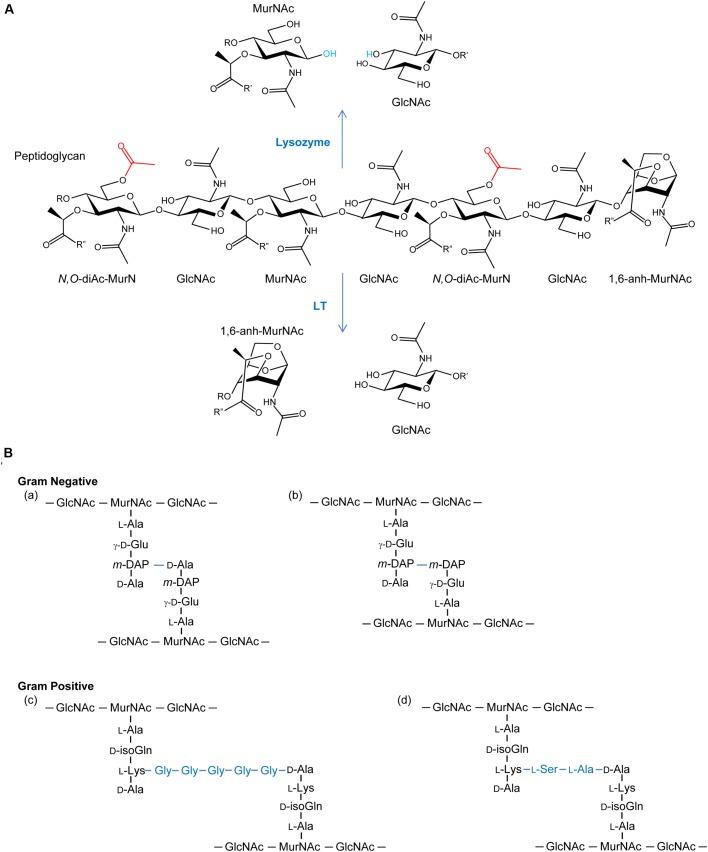
**(A)** Peptidoglycan as a substrate for lysozymes and lytic transglycosylases. Both lysozymes and lytic transglycosylases (LT) cleave PG between MurNAc and GlcNAc residues but their reaction products are different. As hydrolases, lysozymes add water (blue) across the original β-1,4 glycosidic linkage, whereas LTs generate 1,6-anhydroMurNAc (1,6-anh-MurNAc) and GlcNAc products. The O-acetylation of the C-6 OH groups of MurNAc residues (depicted in red) generating *N,O*-di-acetyl-muramoyl products inhibits activity of both lytic enzymes. R, R′, and R^′′^ denote GlcNAc, MurNAc, and stem peptides, respectively. **(B)** Examples of stem peptides and modes of crosslinking. The stem peptides associated with Gram-negative PG typically involve γ-D-glutamic acid (γ-D-Glu) and *meso*-diaminopimpelic acid (*m*-DAP) at the second and third positions, respectively (where the peptide bond between the two involves the γ-carboxyl group of the Glu), from the lactyl moiety of MurNAc. Crosslinking (blue lines) occurs predominately between the *m*-DAP of a PG monomeric unit and the penultimate D-Ala residue of neighboring unit (a), but linkages between two *m*-DAP residues (b) also occur. A greater variation in stem peptide composition is found in the PG from Gram-positive bacteria, but typically isoglutamine (isoGln) and L-Lys occur at positions two and three, respectively. As with Gram-negative bacteria, crosslinking can be direct, but bridging peptides (blue) are often present, such as the pentaglycyl (c) and Ser-Ala (d) peptides associated with the PG in *S. aureus* and *S. pneumoniae*, respectively.

As a key component of bacterial cell walls, peptidoglycan (PG; also known as murein) counters the turgor pressure of the cytoplasm to maintain cell viability. This essential feature, together with its uniqueness to bacteria, presents an “Achilles heel” that has been exploited both naturally and clinically. Thus, in addition to antibiotics that target PG metabolic events, such as the β-lactams and glycopeptides (e.g., vancomycin), PG is the initial target of the innate immune system. Specifically, lysozymes are produced (Ragland and Criss, 2017) to hydrolyze glycosidic linkages between the repeating amino sugar units that form the glycan backbone of PG (**Figure 1**). The initial PG fragments released serve to activate further immune responses (Shimada et al., 2010; Sorbara and Philpott, 2011) while the continued lytic action results in cell rupture and death. This lysozyme response is effective against both Gram-positive and Gram-negative bacteria despite the fact that the PG sacculus of the latter organisms is “protected” by an outer membrane; other factors of the innate immune system, such as lactoferrin (Ellison and Giehl, 1991) and defensins (Hancock and Scott, 2000), disrupt the outer membrane to facilitate exposure of PG to lysozyme. However, many pathogenic bacteria defend against this innate immunity by chemically modifying their PG through O-acetylation. As such, the enzymes involved in the O-acetylation of PG may represent a new opportunity for antibiotic discovery.

This review presents the current understanding of the structure and function relationship of the enzymes that catalyze the O-acetylation and de-O-acetylation of PG. This information thus presents a foundation for the identification of inhibitors that may prove to be leads for one or more novel classes of antibacterials.

## Peptidoglycan Structure And Biosynthesis

### General Structure of Peptidoglycan

The general structure of PG is composed of conserved repeating units of GlcNAc and MurNAc residues joined by β-(1,4)-glycosidic linkages (**Figure 1A**). These chains vary in length, depending on species, and adjacent strands are covalently cross-linked through stem peptides that are associated with the MurNAc residues. Over 50 unique chemotypes of PG are known (Schleifer and Kandler, 1972), which are defined by the composition of the stem peptides and the nature of their crosslinking (**Figure 1B**). Despite this, nascent PG precursors always contain MurNAc with a core pentapeptide stem where the first four stem peptide residues canonically alternate between D- and L-isomers. The first amino acid is attached to the C-3 lactyl moiety of MurNAc and is always D-Ala. At the second position, L-Glu is most often found. However, some bacteria have the ability to amidate this residue (iso-L-Gln); this amidation is catalyzed by an amidotransferase on the synthesized PG precursor (Figueiredo et al., 2012; Münch et al., 2012). The residue at the third position is the most variable, but it is commonly occupied by either L-Lys or *meso*-diaminopimelic acid (*m*DAP), followed by two D-Ala residues at the fourth and fifth positions. The type of crosslink between stem peptides also varies and may occur either directly between the third and fourth positions of the two peptides or through a peptide bridge, such as the penta-Gly and Ser-Ala branches commonly seen in *S. aureus* and *S. pneumoniae*, respectively.

### Biosynthesis of Peptidoglycan

**FIGURE 2:**
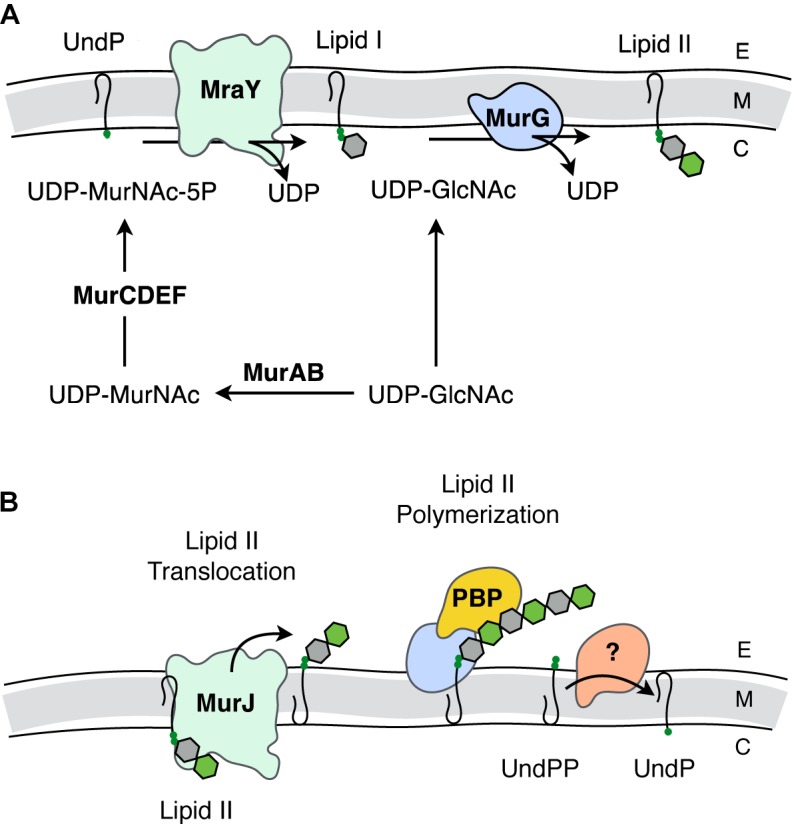
Biosynthesis of PG. **(A)** Intracellular events - biosynthesis of Lipid II. The synthesis of Lipid II (UndPP-MurNAc-pentapeptide-(β-1,4)-GlcNAc) begins with the conversion of UDP-GlcNAc into UDP-MurNAc followed by the addition of amino acids to form the stem pentapeptide (5P). MurNAc-5P is then transferred to the membrane-associated carrier undecaprenyl phosphate (UndP) to generate Lipid I. A second UDP-GlcNAc provides the GlcNAc residue leading to Lipid II production. **(B)** Translocation of Lipid II and extracellular events – polymerization of PG. Lipid II is translocated across the cytoplasmic membrane by MurJ for polymerization of the glycan component by the class I penicillin-binding proteins (PBPs) and/or monofunctional transglycosylases, followed by crosslinking between neighboring stem peptides catalyzed by the class II PBPs. Undecaprenyl pyrophosphate phosphatase(s) hydrolyze UndPP to release a phosphate thereby regenerating UndP. How UndP is flipped back to the cytoplasmic face of the membrane remains unknown. E, extracellular space; M, cytoplasmic membrane; C, cytoplasm.

The biosynthesis of PG can be divided into two major events, the preparation of precursor units within the cytoplasm (**Figure 2A**) and their subsequent polymerization into the existing extracytoplasmic sacculus (**Figure 2B**; reviewed in Egan et al., 2015). The nexus of these activities is the translocation of the lipid-linked PG precursor known as Lipid II.

Lipid II biosynthesis begins with the conversion of the primary metabolite fructose-6-phosphate to uridine diphosphate (UDP)-GlcNAc in three enzymatic steps. The committed PG metabolite, UDP-MurNAc, is then made through the sequential action of MurA and MurB (Marquardt et al., 1992; Benson et al., 1993; Brown et al., 1995). This is followed by the addition of the stem pentapeptide to the C-3 lactyl group of UDP-MurNAc through an enzymatic cascade involving MurC (Liger et al., 1995), MurD (Pratviel-Sosa et al., 1991), MurE (Michaud et al., 1990), and MurF (Michaud et al., 1990). The product UDP-MurNAc-5P is the substrate for MraY, which ligates the muropeptide portion to the C-55 isoprenoid lipid carrier, undecaprenyl phosphate (UndP; bactoprenol) with the release of UMP (Ikeda et al., 1991). The product Lipid I (UndPP-MurNAc-5P), localized on the cytoplasmic face of the cytoplasmic membrane, is ligated to the GlcNAc of a second UDP-GlcNAc with the release of UDP through the action of the glycosyltransferase MurG generating Lipid II (Ikeda et al., 1992).

The question of how Lipid II is translocated across the membrane was a matter of great debate. The controversy involved two opposing hypotheses, with each suggesting that either MurJ (Ruiz, 2008) or FtsW (Mohammadi et al., 2011) is the Lipid II translocase (“flippase”). Both proteins perform non-redundant and essential roles. However, a preliminary report from the Bernhardt and Walker labs (Taguchi et al., 2018) identifies FtsW as a PG polymerase that becomes activated once bound to its cognate penicillin-binding protein (PBP). The PBPs are the enzymes involved in the late steps of PG biosynthesis as they polymerize Lipid II on the extracytoplasmic face of the cytoplasmic membrane to produce the nascent glycan chains necessary for (out)growth of the sacculus. Although this is an area of ongoing research, the long-standing model has been that this assembly process is dependent on both monofunctional glycosyltransferases and the PBPs, specifically those that polymerize glycan chains and then catalyze the cross-linking of neighboring strands to produce the mature sacculus (Goffin and Ghuysen, 1998).

Bacteria produce multiple PBPs, each playing unique roles in PG biosynthesis. In rod shaped cells, some PBPs are recruited to the site of cell division, while others function to elongate the cell. These PBPs are part of multi-protein complexes known as the divisosome and elongasome, respectively (Höltje, 1998; Naninga, 1991). These discrete complexes are organized by the filamentous actin-like cytoskeletal proteins MreB and FtsZ, respectively (Eraso and Margolin, 2011; Fleurie et al., 2014). The biosynthetic complexes also include autolysins, enzymes that lyse PG to provide new sites for sacculus expansion and cell division, as well as for the insertion of transport and secretion systems, pili, and flagella through the PG sacculus.

### Autolysins

The enzymes that lyse PG are glycosidases, amidases, or endopeptidases (recently reviewed in Vollmer et al., 2008). The major glycosidases produced by Gram-positive bacteria for PG metabolism are typically the β-*N*-acetylglucosaminidases. These enzymes hydrolyze the β-1,4 linkage specifically between GlcNAc and MurNAc residues. The only confirmed β-*N*-acetylglucosaminidase reported in Gram-negative bacteria is FlgJ of *Salmonella enterica* where it is responsible for the insertion of flagella (Herlihey et al., 2014). Lysis of the β-1,4-glycosidic linkage between MurNAc and GlcNAc residues is mediated by two distinct enzyme classes, the muramidases (lysozymes) and the lytic transglycosylases (LTs) (**Figure 1A**). Despite the identical substrate specificity however, bacteria only produce the LTs for PG metabolism, and they represent the major glycosidase produced for this purpose by Gram-negative bacteria. Muramidases, on the other hand, are predominantly produced by eukaryotes as defensive bacteriolytic agents as components of their innate immunity systems (Irwin et al., 2011; Ragland and Criss, 2017). The fundamental difference between these two glycosidases is their mechanism of action and consequent reaction products. Whereas the muramidases are hydrolases that release PG fragments with reducing and non-reducing termini of MurNAc and GlcNAc residues, respectively, the LTs are lyases that generate products terminating with 1,6-anhydroMurNAc and GlcNAc residues (Höltje et al., 1975; **Figure 1A**). LTs appear to be ubiquitous in Gram-negative bacteria, and they are utilized in a wide array of physiological processes (reviewed in Scheurwater et al., 2008). LTs are also present in Gram-positive bacteria, though they are not known to be significant contributors to the autolytic profiles in most species. However, they have been identified to participate significantly in the PG lytic events required for endospore germination (Gutelius et al., 2014).

### Discovery and Relevance of PG O-Acetylation

PG is O-acetylated at the C-6 hydroxyl group of MurNAc (**Figure 1A**), a modification that sterically inhibits the productive binding of lysozyme (Clarke et al., 2002; Pushkaran et al., 2015), while also precluding the action of LTs. First discovered in species of *Streptococcus* and *Micrococcus* 60 years ago (Abrams, 1958; Brumfitt et al., 1958), this PG modification is now known to occur primarily in pathogenic species of both Gram-positive and Gram-negative bacteria (**Table 1**). For example, only pathogenic species of the staphylococci, which includes *S. aureus*, O-acetylate their PG and each is highly resistant to human lysozyme (Bera et al., 2006). By contrast, the non-pathogenic staphylococci do not produce O-acetylated PG and they are thus sensitive to lysozyme. Notable pathogens that do not O-acetylate their PG involve some of the Enterobacteriaceae and Pseudomonads, including *Escherichia coli* and *Pseudomonas aeruginosa* (Clarke, 1993; Clarke et al., 2010), and *Bacillus anthracis* (Sychantha et al., 2018).

**Table 1:** Bacterial species (known and hypothetical) that produce O-acetylated PG.

Gram-positive (OatA)		Gram-negative (PatA/PatB and Ape)^1^	
*Staphylococcus aureus*	*Paenibacillus aquistagni*	*Neisseria gonorrhoeae*	*Photorhabdus asymbiotica*
*Staphylococcus capitis*	*Paenibacillus dendritiformis*	*Neisseria animalis*	*Photorhabdus luminescens*
*Staphylococcus carnosus*	*Paenibacillus larvae*	*Neisseria canis*	*Photorhabdus temperata*
*Staphylococcus epidermidis*	*Paenibacillus macquariensis*	*Neisseria cinerea*	*Xenorhabdus bovienii*
*Staphylococcus haemolyticus*	*Paenibacillus polymyxa*	*Neisseria flavescens*	*Xenorhabdus nematophila*
*Staphylococcus hominis*	*Paenibacillus* sp.	*Neisseria lactamica*	*Xenorhabdus poinarii*
*Staphylococcus lugdunesis*	*Paenibacillus uliginis*	*Neisseria macacae*	*Moraxella bovis*
*Staphylococcus pasteuri*	*Lactobacillus acidophilus*	*Neisseria meningitidis*	*Moraxella canis*
*Staphylococcus saprophyticus*	*Lactobacillus amylolyticus*	*Neisseria mucosa*	*Moraxella caprae*
*Staphulococcus* sp.	*Lactobacillus apis*	*Neisseria perflava*	*Moraxella catarrhalis*
*Staphylococcus warneri*	*Lactobacillus bombicola*	*Neisseria polyasacchareal*	*Moraxella cuniculi*
*Macrococcus caseolyticus*	*Lactobacillus brevis*	*Neisseria shayeganii*	*Moraxella equi*
*Listeria grayi*	*Lactobacillus bucheri*	*Neisseria sicca*	*Moraxella lacunata*
*Listeria monocytogenes*	*Lactobacillus casei*	*Neisseria subflava*	*Moraxella nonliquefaciens*
*Listeria seeligeri*	*Lactobacillus crispatus*	*Chromobacterium violaceum*	*Moraxella oblonga*
*Listeria welshimeri*	*Lactobacillus delbrueckii*	*Eikenella corrodens*	*Moraxella pluranimalium*
*Bacillus* sp.	*Lactobacillus jensenii*	*Kingella kingae*	*Campylobacter avium*
*Bacillus amyloliquefaciens*	*Lactobacillus jensenii*	*Kingella oralis*	*Campylobacter coli*
*Bacillus cereus*	*Lactobacillus gasseri*	*Citrobacter youngae*	*Campylobacter cuniculorum*
*Bacillus endophyticus*	*Lactobacillus helveticus*	*Cronobacter sakazakii*	*Campylobacter gracilis*
*Bacillus filamentosus*	*Lactobacillus johnsonii*	*Dickeya dadantii*	*Campylobacter helveticus*
*Bacillus licheniformis*	*Lactococcus lactis*	*Dickeya zeae*	*Campylobacter jejuni*
*Bacillus megaterium*	*Lactobacillus lindneri*	*Morganella morganii*	*Campylobacter upsaliensis*
*Bacillus mycoide*	*Lactobacillus mucosae*	*Proteus hauseri*	*Helicobacter bilis*
*Bacillus pseudomycoides*	*Lactobacillus paracsei*	*Proteus mirabilis*	*Helicobacter cinaedi*
*Bacillus pumilus*	*Lactobacillus reuteri*	*Proteus penneri*	*Helicobacter fennelliae*
*Bacillus subtilis*	*Lactobacillus rhamnosus*	*Proteus vulgaris*	*Helicobacter hepaticus*
*Bacillus thuringiensis*	*Lactobacillus ruminis*	*Cosenzaea myxofaciens*	*Helicobacter jaachi*
*Bacillus velezensis*	*Lactobacillus sakei*	*Providencia alcalifaciens*	*Helicobacter japonicus*
*Bacillus weihenstephanensis*	*Lactobacillus salivarius*	*Providencia urhodogranariea*	*Helicobacter marmotae*
*Brevibacillus* sp.	*Lactobacillus ultunensis*	*Providencia heimbachae*	*Helicobacter mustelae*
*Eggerthella lenta*	*Enterococcus casseliflavus*	*Providencia rettgeri*	*Helicobacter muridarum*
*Lysinibacillus fusiformis*	*Enterococcus faecium*	*Providencia rustigianii*	*Helicobacter magdeburgensis*
*Lysinibacillus sphaericus*	*Enterococcus faecalis*	*Providencia sneebia*	*Helicobacter pylori*
*Lysinibacillus* sp.	*Streptococcus anginosus*	*Providencia stuartii*	*Helicobacter* sp.
*Gemella haemolysans*	*Streptococcus gordonii*		*Helicobacter trogontum*
*Exiguobacterium* sp.	*Streptococcus intermedius*		
*Exiguobacterium sibiricum*	*Streptococcus mitis*		
*Alicyclobacillus montanus*	*Streptococcus oralis*		
*Alicyclobacillus vulcanalis*	*Streptococcus parasanguinis*		
*Viridibacillus* sp.	*Streptococcus pneumoniae*		
*Serinibacter salmoneus*	*Streptococcus pseudopneumoniae*		
*Jatrophihabitans* sp.	*Streptococcus pyogenes*		
*Clostridium amylolyticum*	*Streptococcus salivarius*		
*Clostridium botulinum*	*Streptococcus suis*		
	*Streptococcus thermophilus*		

*^1^Whereas hypothetical homologs of PatA and/or PatB appear to be encoded in the genomes of other Gram-negative bacteria, listed are only those encoded in OAP clusters which include the coding gene for Ape.*

As a non-stoichiometric modification, the extent of PG O-acetylation varies with species, strain, and the age of a culture. Ranging between 20 and 70% (relative to MurNAc concentration) (Clarke and Dupont, 1991; Clarke, 1993), increases in PG O-acetylation by 10-40% can occur when cells enter stationary phase (Pfeffer et al., 2006). It should be noted that *Lactobacillus plantarum* also O-acetylates its PG-associated GlcNAc residues. This modification serves to control its major autolysin, an *N*-acetylglucosaminidase, but it does not influence lysozyme sensitivity (Bernard et al., 2011).

## Pathobiology Of O-Acetylated PG

The resistance of PG to lysozyme digestion conferred by O-acetylation has long been known to have serious implications for human health. Early studies showed that large molecular weight fragments of PG persist in the circulatory systems of hosts to induce a variety of pathobiological effects, including complement activation, pyrogenicity, and arthritogenicity (reviewed in Seidl and Schleifer, 1985). The direct relationship between these effects and PG O-acetylation was clearly demonstrated in a series of animal model studies conducted in the 1980s with *S. aureus* and *N. gonorrhoeae* (reviewed in Clarke et al., 2010). More recent developments at the molecular level demonstrate the critical role that PG O-acetylation plays in the pathogenesis of *S. aureus*-mediated septic arthritis (Baranwal et al., 2017). Also, it has been shown that O-acetylated *S. aureus* PG inhibits production of the important cytokine IL-1β, while concomitantly rendering the bacterium resistant to lysozyme killing in macrophages (Shimada et al., 2010). Furthermore, this modification to PG limits helper T-cell priming thereby permitting reinfection by this pathogen (Sanchez et al., 2017). Similar studies demonstrating escape from the immune response and survival within macrophages due to the O-acetylation of PG have been made with *Streptococcus iniae* (Allen and Neely, 2012), *Listeria monocytogenes* (Aubry et al., 2011), *Helicobacter pylori* (Wang et al., 2012), and *Neisseria meningitidis* (Veyrier et al., 2013). The potential of PG O-acetylation as a viable antivirulence target is underscored by the direct correlation that has been observed between decreased pathogenicity and increased susceptibility of PG to host lysozymes resulting from decreased O-acetylation levels with *S. aureus* (Bera et al., 2005, 2006), *S. pneumoniae* (Davis et al., 2008), *S. iniae* (Allen and Neely, 2012), *Streptococcus suis* (Wichgers Schreur et al., 2012), *Enterococcus faecalis* (Hébert et al., 2007; Le Jeune et al., 2010), *L. monocytogenes* (Aubry et al., 2011; Burke et al., 2014), *H. pylori* (Wang et al., 2012), and *N. meningitidis* (Veyrier et al., 2013). With each of these studies, the enzyme catalyzing PG O-acetylation and/or its regulator was identified as the virulence factor responsible for the decreased pathogenicity observed. In addition to providing increased resistance to lysozyme, the action of the *O*-acetyltransferase in *S. pneumoniae* has been shown to attenuate resistance to β-lactam antibiotics (Crisóstomo et al., 2006).

## Pathways For PG O-Acetylation

Until recently, little was known about the process of PG O-acetylation. Earlier microbiological and biochemical experiments identified it is a maturation event; O-acetylated Lipid II was not found in any organism known to produce *O*-acetyl-PG (Clarke and Dupont, 1991). With *N. gonorrhoeae*, the percentage of O-acetylation of newly synthesized PG was observed to rapidly increase following incorporation of Lipid II into the sacculus, consistent with the notion that PG O-acetylation is a post-synthesis modification (Dougherty, 1983). Subsequent pulse-chase experiments involving *N. gonorrhoeae* (Lear and Perkins, 1983, 1986, 1987), *P. mirabilis* (Gmeiner and Kroll, 1981; Gmeiner and Sarnow, 1987), and *S. aureus* (Snowden et al., 1989) revealed a close temporal relationship between the cross-linking of newly incorporated PG strands and O-acetylation. In each case, an initial burst of PG cross-linking was immediately followed by O-acetylation. The extent of the two events then appeared to gradually rise concomitantly to their final maximal levels over the remainder of the cell cycle. The correlation between transpeptidation and O-acetylation was further demonstrated using sub minimum inhibitory concentrations (MIC) of penicillin G. With all three species, O-acetylation values sharply declined following administration of the penicillin (Martin and Gmeiner, 1979; Blundell and Perkins, 1981; Burghaus et al., 1983; Dougherty, 1985a; Sidow et al., 1990). A correlation of the PBP profiles of penicillin-resistant and susceptible strains of *N. gonorrhoeae* with their respective levels of PG O-acetylation suggested that PBP2 may have a role in mediating the two processes (Dougherty, 1983, 1985b).

**FIGURE 3:**
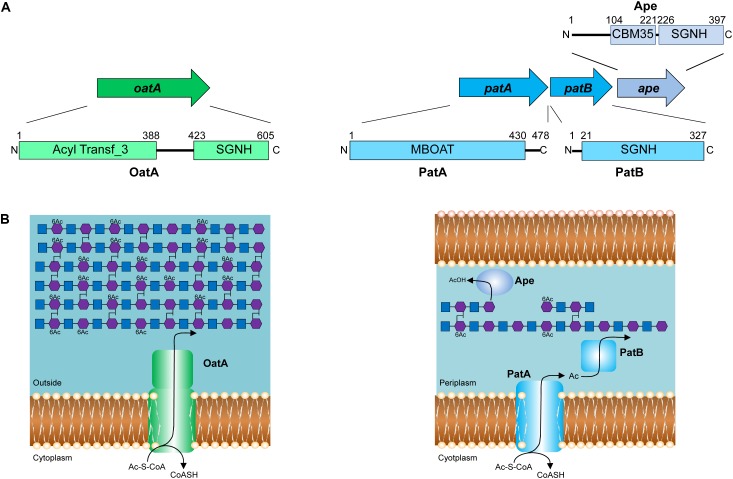
Pathways for the O-acetylation of PG. **(A)** Genetic organization and domain structure of the enzymes involved in PG O-acetylation (OatA, PatA/B) and de-O-acetylation (Ape). **(B)** Proposed models for the O-acetylation of PG in Gram-positive and Gram-negative bacteria. The membrane-spanning N-terminal domain of OatA is thought to translocate acetyl groups across the cytoplasmic membrane from the cytoplasm (presumably provided by acetyl-CoA) for their transfer to PG by the C-terminal *O*-acetyltransferase domain. With Gram-negative bacteria, these two distinct activities are catalyzed by separate proteins, PatA and PatB, respectively. Again, acetyl-CoA is the presumed source of acetyl groups. Ape is produced by these bacteria and localized to the periplasm for removal of the O-acetylation as required for continued PG metabolism.

Identification of the enzymes involved in PG O-acetylation in the Gram-negative bacteria remained unknown for two decades until the availability of bacterial genomes in 2005, when Weadge and Clarke (2005) identified the existence of an AlgI paralog from *P. aeruginosa* within the chromosome of *N. gonorrhoeae*; AlgI is a member of the membrane-bound *O*-acyltransferase (MBOAT) family that functions as part of a multi-component system to acetylate the exopolysaccharide alginate (Franklin and Ohman, 2002). The AlgI paralog was found within a three-gene operon (named OAP for *O*-acetyl-PG) (**Figure 3A**) and knowing that *N. gonorrhoeae* does not produce alginate, the gene product was proposed to function as a PG *O*-acetyltransferase (Weadge and Clarke, 2005). Subsequent deletion mutants of the gene encoding this AlgI homolog in *N. gonorrhoeae*, initially named *pacA*, confirmed that it was important for the production of *O*-acetyl-PG (Dillard and Hackett, 2005). Immediately downstream of *pacA* is a gene encoding a protein of the SGNH/GDSL hydrolase family of esterases (**Figure 3A**). It was recognized to be genetically linked to *pacA*, and hence, it was initially named *O*-acetylpeptidoglycan esterase (*ape2*) (Weadge and Clarke, 2005). However, despite its amino acid sequence similarity with the SGNH family of esterases, Ape2 was later demonstrated to function as a PG *O*-acetyltransferase both *in vivo* and *in vitro* (Moynihan and Clarke, 2010; Veyrier et al., 2013; Ha et al., 2016), and consequently renamed as PatB; PacA was renamed PatA in keeping with the convention for naming acetyltransferases. Downstream of *patB*, a third gene, named *ape1*, was also identified (**Figure 3A**). The protein encoded by *ape1* also belongs to the SGNH/GDSL hydrolase family, and its activity was confirmed to be that of an authentic *O*-acetyl-PG esterase (Weadge and Clarke, 2006).

As a post-synthetic modification, PG O-acetylation occurs in the periplasm of Gram-negative cells (or on the outer surface of the cytoplasmic membrane of Gram-positive bacteria) (reviewed in Clarke and Dupont, 1991). Given the genetic organization of the encoding genes coupled with the demonstrated activities (Dillard and Hackett, 2005; Moynihan and Clarke, 2010), PatA and PatB have been proposed to function in concert as a two-component system (Moynihan and Clarke, 2010; **Figure 3B**). PatA, an integral membrane protein, is thought to function as a membrane transporter, translocating acetyl groups to the periplasm from a cytoplasmic source, presumably acetyl-CoA. Localized to the periplasm (Moynihan and Clarke, 2010), PatB would then transfer the acetyl groups to the C-6 hydroxyl groups of MurNAc residues in PG. Whether the acetyl group is transferred between these two proteins directly, or through an acetyl carrier molecule is not known.

Almost concurrently with the discovery of the genes for PG O-acetylation in *N. gonorrhoeae*, Bera et al. (2005) identified *O*-acetyltransferase A (OatA) as the enzyme responsible for PG O-acetylation in Gram-positive bacteria. Since then, homologs of OatA from several other Gram-positive bacteria have been characterized, including those from clinical isolates of *S. pneumoniae* (Crisóstomo et al., 2006), *E. faecalis* (Hébert et al., 2007), *L. plantarum* (Weadge and Clarke, 2005; Bernard et al., 2011), and *L. monocytogenes* (Aubry et al., 2011).

OatA is predicted to be bi-modular, being comprised of an N-terminal integral membrane domain (OatA_N_) and a C-terminal extracytoplasmsic domain (OatA_C_) (**Figure 3B**). Interestingly, OatA_N_ belongs to the Acyltransferase 3 (AT3) family of proteins and not the MBOATs. This AT3 domain is linked to OatA_C_ which, like PatB, is a member of the SGNH/GDSL hydrolase family (Bernard et al., 2011, 2012; Sychantha et al., 2017). Thus, despite the difference in protein family membership of the membrane-spanning OatA_N_, the overall domain architecture of OatA resembles the two-component PG O-acetylation system of Gram-negative bacteria. Like PatA, OatA_N_ is proposed to translocate acetyl groups across the cytoplasmic membrane for the surface-exposed OatA_C_ to function like PatB as the *O*-acetyltransferase (**Figure 3B**). Unlike the Gram-negative system, however, OatA is not known to be coupled with an Ape-like *O*-acetyl-PG esterase. This may be a reflection of the fact that the major autolysins of Gram-positive bacteria are not LTs, which require a free C-6 hydroxyl group on MurNAc residues for activity, but instead are mainly peptidases and glucosaminidases that are not affected by the modification.

## Structure And Function Relationship Of PG *O*-Acetyltransferases

### Activity and Specificity of *O*-Acetyltransferases

**FIGURE 4:**
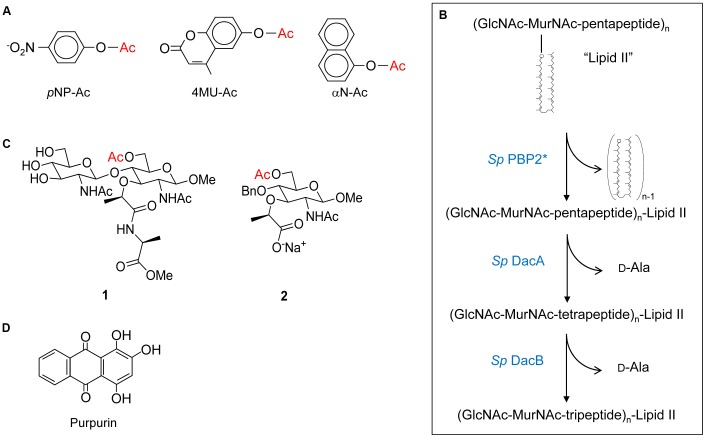
Structures of substrates and inhibitors of PG *O*-acetyltransferases and *O*-acetylesterases. **(A)** Chromogenic *p*-nitrophenyl acetate (*p*NP-Ac) and fluorogenic 4-methylumbelliferyl acetate (4MU-Ac) and α-naphthyl acetate (αN-Ac) as substrates for detection of esterase activity. **(B)** Production of Lipid II-based polymers as substrates for *O*-acetyltransferase activity. Polymerization of Lipid II by PBP2 from *S. pneumoniae* produces homopolymers of GlcNAc and MurNAc with associated pentapeptides, and the concomitant release of all but the terminal bactoprenol (undecaprenyl) residues. Successive hydrolytic reactions with the carboxypeptidases DacA and DacB (both also from *S. pneumoniae*) generate homopolymers with associated tetrapeptides and tripeptides, respectively. **(C)** Structures of synthetic *N,O*-diAc-MurN-based substrates (compounds 1 and 2) for analysis of Ape activity. **(D)** Structure of the Ape inhibitor purpurin.

Despite their discovery over a decade ago, it was not until recently that the biochemical details of PG *O*-acetyltransferases were delineated. The complexities of determining the specificity and kinetic parameters of these membrane-associated enzymes were compounded by the lack of available PG-based substrates; attempts to isolate useful quantities of homogeneous muropeptide fractions from natural PG is futile. This changed when it was discovered that these enzymes recognized surrogate compounds as donor and acceptor substrates. Thus, a convenient assay utilizing chromogenic or fluorometric acetyl donors (**Figure 4A**) and GlcNAc-based acceptor substrates was developed (Moynihan and Clarke, 2013), which has been used to kinetically analyze both PatB (Moynihan and Clarke, 2013, 2014a) and OatA (Sychantha et al., 2017; Sychantha and Clarke, 2018). Both enzymes were found to be very weak esterases compared to authentic PG *O*-acetylesterase Ape. Nonetheless, this activity was exploited to determine the biochemical properties of the enzymes, in addition to the steady-state (Sychantha et al., 2017; Moynihan and Clarke, 2014a,b) and, in the case of *S. pneumoniae* OatA_C_, pre-steady kinetic parameters (Sychantha and Clarke, 2018). These studies revealed the dependence of the hydrolytic reaction on pH with the p*K*_a_ of an essential ionizable group between 6.25–6.4 and 6.9–7.3 for *Ng*PatB (Moynihan and Clarke, 2014a) and *Sp*OatA_C_ (Sychantha and Clarke, 2018), respectively, consistent with the participation of a His residue. Neither enzyme is inhibited by metal chelators indicating that they are not metallo-enzymes. However, catalysis of hydrolysis by both is inhibited by the mechanism-based inhibitor methanesulfonyl fluoride indicating the involvement of a nucleophilic Ser residue.

Both PatB and OatA are able to utilize chitooligosaccharides as acceptor substrates. Increases in catalytic efficiency are seen with increasing length of oligosaccharide suggesting a binding cleft comprised of subsites (Moynihan and Clarke, 2014a; Sychantha et al., 2017). However, these increases are relatively small suggesting that the subsites binding MurNAc residues also accommodate elements of their associated stem peptides, which of course are not present in the chitooligosaccharides. This postulate of stem peptide involvement in substrate binding and recognition, at least for OatA_C_, was borne out in a study employing a PG-based substrate produced through the polymerization of the PG biosynthetic precursor Lipid II (Sychantha et al., 2017). The PBP-catalyzed transglycosylation of Lipid II provided a pool of homopolymers of GlcNAc-MurNAc-pentapeptide with degrees of polymerization between 2 and 12 (**Figure 4B**). Subsequent treatment of these pools with the carboxypeptidases DacA and then DacB provided homopolymers with tetrapeptide and tripeptide stems, respectively. Using the three distinct pools of PG homopolymers, *Sa*OatA_C_ was shown to have high specificity for only those with pentapeptide stems. *Sp*OatA_C_, on the other hand, is incapable of using the pentapeptide-based oligomers as substrate. Instead, it has specificity for those with tetrapeptide stems (Sychantha et al., 2017). Having such distinct specificities has implications regarding the timing of the O-acetylation process in these two Gram-positive human pathogens. As PG crosslinking and any subsequent carboxypeptidase activity of the PBPs generates tetrapeptide stems, the specificity of *Sa*OatA_C_ for muroglycans with pentapeptide stems implies O-acetylation would have to follow immediately after the transglycosylation of the Lipid II precursor into the existing PG sacculus. Furthermore, such a close temporal association of these respective activities implies that *Sa*OatA would need to be physically associated with the transglycosylase(s) involved in *S. aureus* PG biosynthesis. The need for this protein-protein association is supported by the observation that *Sa*OatA_C_ only weakly binds to PG (Kell, 2016).

Steady-state kinetic analyses of the *O*-acetyltransferase activities of *Ng*PatB and *Sp*OatA_C_ using chromogenic acetyl donors and chitooligosaccharide acceptors revealed that both enzymes follow a ping-pong bi-bi mechanism of action (Moynihan and Clarke, 2014b; Sychantha and Clarke, 2018). In this two-step process, the enzymes first bind the acetyl donor molecule and transfer the acetyl moiety to a catalytic residue forming a covalent adduct while releasing the first product. With the chromogenic substrates, the chromogen would be this first product. Its release is followed by the binding of the acceptor substrate to the acetylated enzyme. The acetyl group is then transferred to the hydroxyl group of the acceptor sugar and the enzyme returns to its resting state with the release of the O-acetylated product. When functioning as an esterase, water simply replaces the sugar as the acceptor “substrate.” However, a follow-up pre-steady-state kinetic analysis of this esterase activity revealed that water does not serve as a very efficient acceptor for the hydrolytic event. Using *Sp*OatA_C_ with *p*-nitrophenyl acetate as substrate, hydrolysis of the reaction intermediate was shown to be the rate-limiting step. The half-life of this intermediate was estimated to be over 70 s (Sychantha and Clarke, 2018), indicating an extremely slow process, and accounting for the overall poor hydrolytic activity of these enzymes. Indeed, acetyl-intermediates of both *Ng*PatB (Moynihan and Clarke, 2014b) and *Sp*OatA_C_ have been trapped (Sychantha and Clarke, 2018) and with both, the acetyl group was observed to be covalently linked to a Ser residue.

### Structure and Mechanism of Action of PG *O*-Acetyltransferases

**FIGURE 5:**
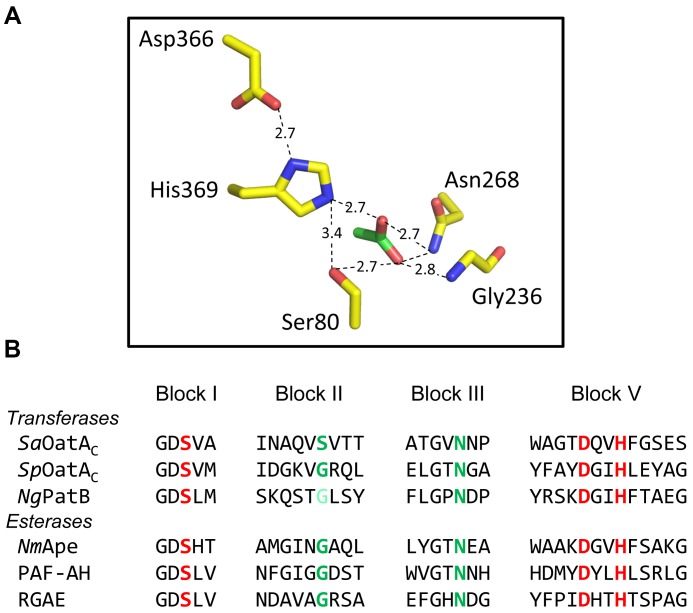
Active site organization and sequence comparison of representative members of the SGNH/GDSL families of enzymes. **(A)** Hydrogen-bonding network of catalytic triad and oxyanion hole residues (yellow) and an acetate (green) at the active site of *Nm*Ape (PDB 4K7J). **(B)** Alignment of residues comprising the signature sequence Blocks of OatA and PatB with representative enzymes of the SGNH/GDSL family with known X-ray crystal structures. Residues in red and green comprise the catalytic triads and oxyanion holes, respectively. The light green Gly of *Ng*PatB denotes the fact that participation of this invariant Gly at the oxyanion hole of PatB has yet to be demonstrated experimentally. PAF-AH, human platelet-activating factor acetyl hydrolase; RGAE, rhamnogalacturonan acetyl esterase (PDB IDs 1BWQ and 1DEX, respectively).

The SGNH family of the GDSL superfamily of esterases/lipases is named for the participation of Ser, Gly, Asn, and His residues in their mechanism of action. The Ser and His comprise a catalytic triad, while all four form the “oxyanion hole,” a region at the catalytic center of the enzymes that stabilizes the putative oxyanion transition states leading to and from the acyl-enzyme intermediate (Mølgaard et al., 2000; **Figure 5A**). The Gly and Asn are found within consensus motifs named Block II and Block III, respectively, while the catalytic Ser and His (and Asp) comprise Blocks I and V, respectively (**Figure 5B**). The Block II residues form a type-II β-turn to direct the backbone NH of the invariant Gly toward the active site so that it can serve together with the backbone NH of the Ser and the side-chain amide of the Asn to stabilize the oxyanion of the putative tetrahedral intermediate. Invariant Ser, His, and Asp residues are present in the respective alignments of PatB and OatA_C_ homologs, and the single replacements of these residues in the enzymes from *N. gonorrhoeae* and both *S. aureus* and *S. pneumoniae*, respectively, abrogates catalytic activity (Moynihan and Clarke, 2014a,b; Sychantha and Clarke, 2018). Also, it is these Ser residues that were found to form adducts to the acetyl groups of the acetyl-intermediates of both *Ng*PatB and *Sp*OatA mentioned above. Invariant Asn residues are also present in both the PatB and OatA_C_ homologs and consistent with their expected role in stabilizing oxyanion transition states, their replacements also leads to loss of *O*-acetyltransferase activity (Moynihan and Clarke, 2014b; Sychantha and Clarke, 2018). Whereas an invariant Gly is observed Block II of all PatB homologs, this Gly is not invariant in OatA_C_ and it is replaced by a Ser in many homologs, including all of those produced by the streptococci (Sychantha and Clarke, 2018; **Figure 5**). However, it should be noted that experimental evidence for the participation of the invariant Gly residue in Block II of PatB has yet to be obtained. As such, the importance of this residue in the transferases compared to the esterases remains to be established.

**FIGURE 6:**
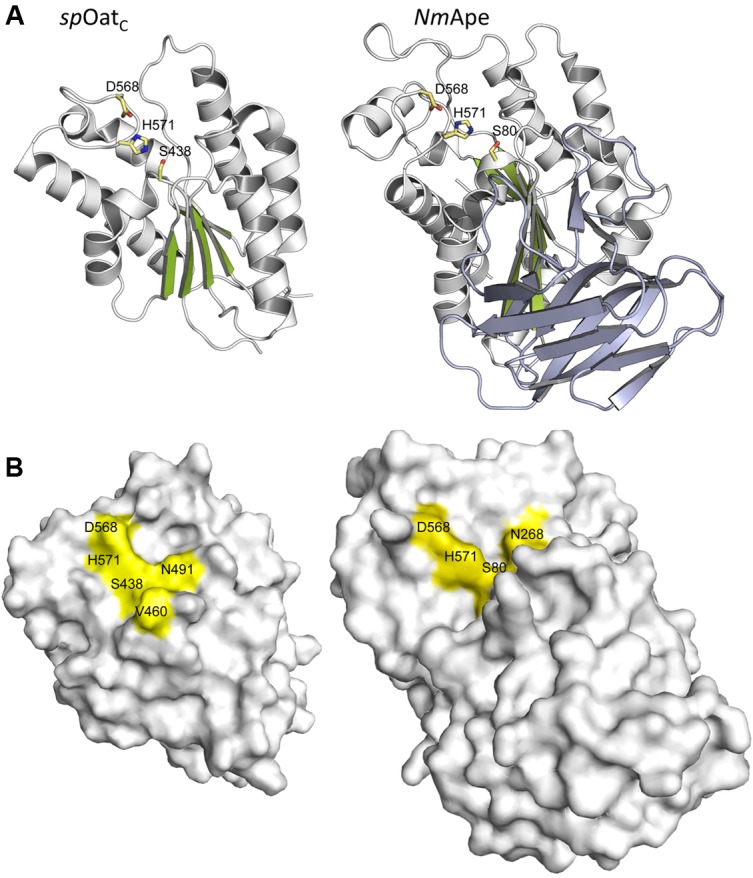
Structures of *Sp*OatA_C_ and *Nm*Ape. **(A)** Cartoon representations identifying the α-helices (gray), β-strands (green), and coils (white) of the atypical α/β hydrolase fold adopted by the two enzymes. Also shown, in stick form, are the amino acids comprising the respective catalytic triads. The β-strands forming the putative CBM of *Nm*Ape are depicted in steel blue. **(B)** Surface representations of *Sp*OatA_C_ and *Nm*Ape depicting the catalytic triad and oxyanion hole residues within shallow pockets of the respective enzymes (PDB IDs 5UFY and 4K7J, respectively).

The only X-ray crystal structure of a PG *O*-acetyltransferase reported is that of *Sp*OatA_C_ (Sychantha et al., 2017). As predicted earlier for *L. plantarum* OatA (Bernard et al., 2011) and *Ng*PatB (Bernard et al., 2012; Moynihan and Clarke, 2014b), the enzyme adopts an atypical α/β hydrolase fold (**Figure 6A**) which most closely resembles the structure of human platelet-activating factor acetylhydrolase (Ho et al., 1999) (PDB ID: 1BWQ) and *E. coli* thioesterase I/protease I/lysophospholipase L1 (Lo et al., 2003) (PDB ID: 1IVN), two other members of the SGNH/GDSL hydrolase superfamily. A core parallel β-sheet of five strands is sandwiched between seven α-helices which form a shallow active site pocket (**Figure 6B**). At its center, the triad of Ser-His-Asp residues is aligned appropriately to serve catalytically, and the invariant Asn is positioned to help stabilize a transition state.

**FIGURE 7:**
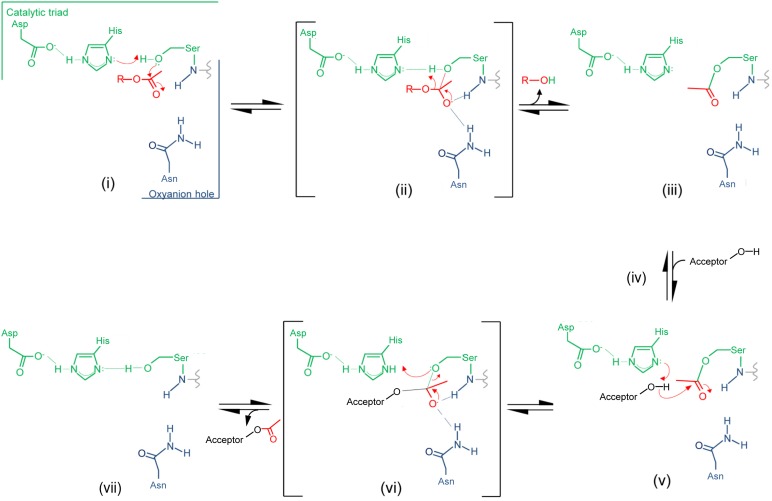
Proposed mechanism of action of PG *O*-acetyltransferases and *O*-acetyl-PG esterases. (i) The reaction is initiated with the binding of an acetyl donor (transferases) or substrate (esterases), depicted in red, into the active site of the respective enzyme. The H-bonding network between the catalytic triad residues increases the nucleophilicity of the catalytic Ser hydroxyl which attacks the carbonyl center of the bound acetyl group. (ii) The putative transient tetrahedral oxyanion intermediate is stabilized by the oxyanion hole residues (backbone amide of the Ser438 and side-chain amide of Asn491 for *Sp*OatA_C_). (iii) An acetyl-enzyme intermediate forms with the departure of the donor group (transferase) or de-acetylated reaction product (esterase). (iv) A MurNAc residue on PG (transferase) or water (esterase) binds, and (v) the catalytic His serves as a base to abstract the proton from either the C-6 hydroxyl group or water to permit its nucleophilic attack of the resulting oxyanion on the carbonyl center of the acetyl-enzyme. (vi) The second transient oxyanion formed is stabilized by the oxyanion hole residues prior to its (vii) collapse releasing either the 6-*O*-acetyl-PG or free acetate product.

Insight provided by the structural studies, together with the understanding of their kinetic behavior and dependence on pH, supports a double-displacement, ping-pong bi-bi reaction as the mechanism of action of the *O*-acetyltransferases, analogous to that of the well-characterized serine esterases. Thus, the alignment and H-bonding network of the catalytic triad (**Figure 6B**) would render the hydroxyl group of the catalytic Ser nucleophilic (**Figure 7**). The deprotonated Oδ of this Ser could attack the carbonyl carbon of the acetyl donor molecule leading to a putative tetrahedral oxyanion transition state, which would be stabilized by the oxyanion hole residues. This oxyanion would rapidly collapse to the covalent acetyl-enzyme intermediate concomitant with His-catalyzed protonation and subsequent release of the donor product. Then, instead of a water molecule associated with esterase activity, a glycan strand PG would bind into the active site cleft and the deprotonated catalytic His would now function as a base to abstract the C-6 hydroxyl proton of an appropriately positioned MurNAc residue rendering it nucleophilic. Attack of this C-6 alkoxide on the carbonyl center of the acetyl-Ser forms a second tetrahedral oxyanion transition state, which is again stabilized by the oxyanion hole residues. Collapse of this transition state leads to the release of the O-acetylated PG.

As noted above, the PG *O*-acetyltransferases have the capacity to function as esterases *in vitro*, at least when provided with activated esters as substrate. However, *in vivo* hydrolytic activity would have to be minimized, if not precluded, so as to limit a futile cycle of acetate uptake and loss involving a valuable cytoplasmic metabolite, presumably acetyl-CoA. With its highly exposed active site on its surface (**Figure 6B**), *Sp*OatA_C_ does not appear to provide a water-limiting environment to help mitigate the hydrolytic event. However, it is possible that such an environment is formed by the juxtaposition of this catalytic domain with its associated N-terminal integral membrane domain and, additionally, with both the cytoplasmic membrane and the insoluble PG sacculus. The opportunity to observe the covalent adduct of the mechanism-based inhibitor methansulfonyl fluoride with the catalytic Ser in the crystal structure of the inactive enzyme provides some further insight into the mechanism of action of OatA, specifically as to how the hydrolytic reaction is indeed minimized. This adduct represents a transition-state analog that mimics the attack of a water molecule on the acetyl-enzyme intermediate during the hydrolytic reaction of esterases (Myers and Jun, 1954). The orientation of the adduct suggests that the approach to the carbonyl C of the bound acetyl group by an acceptor ligand (e.g., water or a carbohydrate) would have to be from a hydrophobic region located at the back of the active site. Thus, the trajectory of a hydrolytic water passing across this hydrophobic region is an unfavorable condition that might explain the very slow half-life of the intermediate observed in the pre-steady-state kinetic experiments described above. A Val or Ile residue exists at position 490 in all aligned OatA homologs. These invariant Val/Ile residues are located at position 5 in the Block II consensus motif and they are not present in other SGNH/GDSL esterase members (**Figure 4**). Interestingly, site-specific replacement of Val490 in *Sp*OatA_C_ with Ala and Gly, the residues present at this same position in the esterases, rhamnogalacturonan acetyl esterase, and platelet-activating factor acetyl hydrolase, respectively, resulted in increased esterase activity and a concomitant 5- and 10-fold decrease in transferase activity, respectively. These observations suggest that, in addition to minimizing the approach of water to an acetyl-OatA intermediate, this hydrophobic region at the active center may assist the binding and positioning of a carbohydrate acceptor, recognizing the existence of their hydrophobic regions. A co-crystal structure of the enzyme bound by a ligand would help resolve this issue, but unfortunately none have been reported to date.

## PG de-O-Acetylation

### PG *O*-Acetylesterases

As discussed above, the O-acetylation of PG controls endogenous autolytic activity associated with PG metabolism (Dougherty, 1983; Strating and Clarke, 2001; Pfeffer et al., 2006; Emirian et al., 2009; Laaberki et al., 2011; Moynihan and Clarke, 2011; Rae et al., 2011; Bonnet et al., 2017), particularly in Gram-negative bacteria, including that required for cell septation/division (Dougherty, 1983; Emirian et al., 2009; Laaberki et al., 2011). In Gram-negative bacteria, the LTs represent the major autolysins involved with PG metabolism and cell septation (Scheurwater et al., 2008). As their catalytic mechanism produces a 1,6-anhydromuramoyl reaction product, clearly the presence of a C-6 *O*-acetyl group would preclude this activity and thereby provide a means of autolytic control at the substrate level. Hence, Gram-negative cells producing *O*-acetylated PG encode an *O*-acetyl-PG esterase (Ape) which removes the blocking acetyl group (**Figure 3B**), thereby permitting continued PG metabolism (Weadge and Clarke, 2006).

Like PatB, Ape also belongs to the SGNH/GDSL superfamily of serine esterases, and homologs align with the acetyl-xylan esterases of carbohydrate esterase 3 (CE3) enzymes of the CAZy database (Carbohydrate-Active enZYmes Database, 1998). All members of the CE3 family have invariant Ser, His, and Asp residues that were postulated to form a catalytic triad, but no mechanistic investigations had been conducted on any of these enzymes prior to the discovery of Ape. A chemical modification and site-directed engineering study on *N. gonorrhoeae* Ape confirmed the essential role of Ser80, His369, and Asp366 in the catalytic mechanism of this enzyme (Weadge and Clarke, 2007), supporting its preliminary identification as a GDS(L/H) serine esterase (Weadge and Clarke, 2006). In a follow-up study, the roles of eight other invariant or highly conserved residues were probed by a kinetic and substrate-binding characterization of enzyme variants possessing site-specific replacements (Pfeffer et al., 2013). Based on these data combined with a structural prediction of the enzyme, Gly236 and Asn268 were proposed to comprise the oxyanion hole that would stabilize transition states formed during the enzyme’s reaction mechanism while Gly78, Asp79, His81, Asn235, Thr267, and Val368 were proposed to position the catalytic residues appropriately and/or participate in substrate binding. The identification of Gly236 and Asn268 as oxyanion hole residues confirmed the classification of Ape as an SGNH esterase and that its mechanism of action follows that of other classical Ser esterases (Pfeffer et al., 2013; **Figure 6**).

As a reflection of the complexities of working with PG as an *in vitro* substrate, and like the studies described above on PatB and OatA, preliminary kinetic information for Ape were obtained using non-carbohydrate-based, synthetic substrate analogs. But a more meaningful analysis of the binding and kinetic properties of the enzyme requires substrates more closely related to PG. With this in mind, two such specific soluble substrates for Ape involving a MurNAc core were designed and synthesized (Hadi et al., 2011). The water-soluble mono- and disaccharide substrate analogs 1 and 2 (**Figure 4C**) were synthesized and then characterized kinetically. Both compounds 1 and 2 serve as substrates for Ape, but the *k*_cat_/*K*_M_ values obtained were approximately 10-fold less than that for highly activated *p*-nitrophenyl acetate. Nonetheless, these data for the hydrolysis of an unactivated acetate ester at the C-6 of a MurNAc in the mono- and disaccharide-based substrates suggest that binding to polymeric PG is not a strict requirement for efficient catalysis. Moreover, the minimal difference in efficiency between the two substrates indicates that the presence of the methyl ester-protected L-Ala attached to the lactyl moiety of MurNAc is not an essential element for substrate recognition. Substitution of the β-1,4-linked GlcNAc residue with an *O*-benzyl ether without significant kinetic consequence also suggests that minimal contacts occur between the enzyme and this amino sugar. However, it is likely that an entropic driving force from movement of the benzyl group from bulk solvent into a relatively hydrophobic binding pocket compensates for any loss of enthalpic interactions with the GlcNAc residue covalently linked to a Ser residue.

### Structure and Mechanism of Action of PG *O*-Acetylesterases

The crystal structure of the highly homologous Ape from *N. meningitidis* (96% identity between *N. gonorrhoeae* and *N. meningitidis* Ape) was solved and revealed some interesting and unexpected features (Williams et al., 2014). The overall fold of the enzyme involves two domains, the catalytic domain comprising residues 45–99 and 227–393, and an intervening β-sandwich domain formed by residues 104–221 (**Figure 6A**). As predicted earlier (Pfeffer et al., 2013), the catalytic domain adopts the canonical α/β-hydrolase fold of the SGNH hydrolase superfamily complete with the catalytic Ser-His-Asp residues aligned proximal to the oxyanion Asn and Gly residues (**Figure 5A**). However, in contrast to other known Ser esterase-like mechanisms, binding of substrate induces a 90° rotation of the Ser side chain to its catalytically competent position. Also, the oxyanion hole Gly residue appears to provide a gate that may close the active site in the absence of substrate. Hence, it appears that Ape, and possibly the other CE3 family enzymes, functions using an unusual substrate-induced catalytic triad.

The substrate-binding groove is open while a tight pocket accommodates the acetyl moiety (**Figure 6B**). These features, together with the rotational flexibility of the catalytic Ser, may account for the relative substrate promiscuity demonstrated by Ape (Weadge and Clarke, 2006, Clarke, 2007). The absence of any other observed contacts with added ligands in the different crystal structures reported is consistent with the kinetic data obtained with the MurNAc-based substrate analogs mentioned above. However, the smaller N-terminal domain formed by residues 104–221 is proposed to represent a carbohydrate-binding module similar to those of the CBM35 family members of the CAZy database (Montanier et al., 2009). The family 35 CBMs are typically associated with bacterial hydrolases with specificity for plant cell wall polysaccharides. Interestingly however, rather than recognizing the substrates of the cognate catalytic domains, as typical of most CBMs, these CBMs are thought to help anchor the hydrolases to the bacterial cell wall through their capacity to bind uronic acid sugars (Montanier et al., 2009). As enzymes secreted to the periplasm, it is possible that the CBM35-like domain of Ape serves to retain it on PG for its localized activity as required for PG metabolism. How such temporal and functional specificity is conferred remains unknown, but it is also equally possible that Ape associates with the protein complexes (elongasomes and divisosomes) proposed to be responsible for cell elongation and division (Höltje, 1998; Naninga, 1991). Such associations would ensure removal of blocking *O*-acetyl groups as required for LT activity and PG growth/remodeling.

### Discovery of Ape Inhibitors

With the production and employment of both PatA/B and Ape, it would appear that the Gram-negative bacteria balance the level of PG O-acetylation to control continued PG metabolism. Recognizing this important role, it was postulated that these enzymes may serve as new targets for antibiotic development (Moynihan and Clarke, 2010, 2013; Weadge and Clarke, 2006). Indeed, Ape activity is required for survival of *N. meningitidis* in animal models (Veyrier et al., 2013) and attempts to generate deletion mutants in *N. gonorrhoeae* failed (Weadge and Clarke, 2006). Also, no strain of a bacterium that normally produces *O*-acetyl-PG has been found to be devoid of the modification. For example, the chromosome of *N. gonorrhoeae* RD5 harbors a truncated *patA* gene (Dillard and Hackett, 2005), but careful analysis of the PG isolated from this strain revealed it retains between 10 and 15% O-acetylation (Rosenthal et al., 1982; Rosenthal et al., 1983; Swim et al., 1983). Hence, it appears that an inhibitor of either the *O*-acetyltransferase and/or *O*-acetylesterase activities would be detrimental to the pathogens that produce *O*-acetyl-PG.

To identify inhibitors that could be used to prove the principle that these enzymes would serve as novel antibiotic targets, a fluorogenic assay for *O*-acetylesterase activity was made amenable for high throughput screening. With 4-methylumbelliferyl acetate as substrate and *N. gonorrhoeae* Ape as the target, an overall *Z*′ score of 0.62 was obtained for the assay, allowing a threshold for screening to be set at 65% residual activity. The assay was tested in a pilot screen and seven compounds were identified as true inhibitors of *Ng*Ape (Pfeffer and Clarke, 2012). Dose-response curves identified five of these compounds with respectable IC_50_ values, which ranged between 0.3 and 23 μM. Of these, purpurin (a red/yellow dye from the madder plant) (**Figure 4D**) was selected for further analysis based on its ready availability and relative solubility. Its inhibition of Ape was determined to be competitive with a *K*_i_ value of 4.8 μM (Pfeffer and Clarke, 2012).

## Future Directions

Irrespective of the carbohydrates they modify, O-acetylation systems for modifying cell wall glycans and exopolysaccharides have been organized into two general categories: the single protein (AT3-dependent) and multi-protein (MBOAT-dependent) pathways (Moynihan and Clarke, 2011; Gille and Pauly, 2012). Of these, the respective transmembrane protein component (AT3 or MBOAT) is postulated to present an acetyl-donor substrate for an SGNH-hydrolase-like enzyme that catalyzes the glycan modification. The only other SGNH hydrolase-like enzymes involved in bacterial cell wall modifications, beyond OatA and PatB, that have been characterized biochemically are *P. aeruginosa* AlgX (Riley et al., 2013) and AlgJ (Baker et al., 2014), and *Bacillus cereus* PatB1 (Sychantha et al., 2018), enzymes responsible for the O-acetylation of alginate and secondary cell-wall polysaccharide in the respective pathogens. As with PG, the importance of cell wall O-acetylation for virulence in bacteria has also called attention to these systems as potential targets for the development of novel therapeutics. To this end, informing future discovery of *O*-acetyltransferase inhibitors and their development into efficacious antibiotics requires a detailed understanding of the structural and functional relationships of more of these enzymes.

A major unanswered question that remains for each of these O-acetylation systems is the nature of the biological source of acetyl group and how it is mechanistically transported to the extracytoplasmic *O*-acetyltransferases. Whereas the associated MBOAT or AT3 proteins have been proposed to serve this function, the precise mechanism is not known. With some AT3 family members, such as the xanthan *O*-acetyltransferases GumF and GumG produced by *Xanthomonas campestris* (Vorhölter et al., 2008) and the trehalose corynomycolate *O*-acetyltransferase TmaT from *Corynebacterium diptheriae* (Yamaryo-Botte et al., 2015), the C-terminal SGNH hydrolase domain is absent. These enzymes modify the respective sugar moiety associated with a lipid carrier on the cytoplasmic face, and they are subsequently translocated across the membrane for further processing. Presumably, acetyl-CoA is used as source of cytoplasmic acetyl group to confer these modifications. Based on the individual activities of AT3 family proteins, it is tempting to speculate that one linked to a catalytic domain with extracytoplasmic function could produce an activated lipid-linked acetyl donor. However, a lipid species of this nature would likely be transient in order to maintain steady-state levels of O-acetylation. Hence, the constant turnover of such a molecule could be a reason why it may have been overlooked or undetected in previous lipidomic studies. Indeed, a similar phenomenon was seen recently with the *O*-acetyl TMCM produced by TmaT (Yamaryo-Botte et al., 2015). Alternatively, an acetyl-lipid may not exist and instead the transmembrane domain of these proteins may function as a channel, moving acetyl groups across the membrane before presenting them to the extracytoplasmic transferases. While there is currently neither evidence nor precedent to support this, preliminary experiments performed by us with *Sp*OatA_C_ and *Sa*OatA_C_ revealed that these proteins can use *O*-acetyltyrosine as a donor (unpublished data). Consistent with this observation, it was found that the predicted surface topology of the transmembrane domain of OatA from both organisms contains numerous conserved tyrosine residues throughout their predicted transmembrane helices, suggesting that a tyrosine relay could be involved. Regardless, a complete understanding of the mechanism of O-acetylation will require examination of the full length structure of OatA and/or PatA. Although X-ray crystallography could be used for these structural determinations, current advances in cryo-electron microscopy may provide an alternative and more informative approach. For example, the structure of hemoglobin (64 kDa) was recently solved at 3.2Å resolution by cryo-EM (Danev and Baumeister, 2017; Khoshouei et al., 2017) and advances with this technology continue. As OatA is a 70-kDa protein and assumed to be monomeric, cryo-EM may represent a feasible approach for its study. Such studies, together with those discussed above, would further useful information as a foundation for drug discovery.

Finally, it remains to be established if inhibiting only PG *O*-acetyltransferases and/or *O*-acetyl-PG esterases with small molecule inhibitors will be sufficient to render bacterial pathogens exploiting these enzymes susceptible to an efficacious immune response. As the C-6 hydroxyl group of muramoyl residues in PG is also the sight for binding of cell wall polymers, such as teichoic acids and the secondary cell wall polysaccharides of Gram-positive pathogens, and lipoproteins of Gram-negative bacteria (Schleifer and Kandler, 1972), it is possible that cells may respond to the challenge of an anti-O-acetylation inhibitor by increasing production of these other modifying materials. Interestingly however, Bera et al. (2007) observed that such does not appear to occur, at least in *S. aureus*, where Δ*tagO* and Δ*oatA* mutants did not produce higher levels of PG O-acetylation or teichoic acids, respectively [TagO is essential for teichoic acid production (Weidenmaier et al., 2004)]. Nonetheless, the double knockout mutant was the most susceptible strain of the three strains to lysis by lysozyme. As it is not clear what other compensatory action these mutants may have made, such as alterations to crosslinking (Atilano et al., 2010), only the discovery of inhibitors and their use in *in vivo* experiments might prove the principle of the drugability of PG O-acetylation systems in this and other important human pathogens.

## Author Contributions

DS, AB, CJ, and AC prepared various sections of the manuscript and reviewed the final draft.

## Conflict of Interest Statement

The authors declare that the research was conducted in the absence of any commercial or financial relationships that could be construed as a potential conflict of interest.
